# Robot assisted stereotactic surgery improves hematoma evacuation in intracerebral hemorrhage compared to frame based method

**DOI:** 10.1038/s41598-025-97738-1

**Published:** 2025-04-11

**Authors:** Lindong Lou, Hongcai Wang, Maosong Chen, Jingbo Zhu, Shiwei Li

**Affiliations:** Neurosurgery Department of Ningbo Medical Center Lihuili Hospital, Ningbo, Zhejiang China

**Keywords:** Intracerebral hemorrhage, Robot-assisted stereotactic surgery, Frame-based stereotactic surgery, Hematoma evacuation, Neurological disorders, Stroke

## Abstract

Intracerebral hemorrhage (ICH) requires prompt hematoma evacuation to mitigate poor outcomes. This study compares robot-assisted stereotactic surgery with traditional frame-based methods for ICH evacuation. A retrospective analysis of 131 patients (45 robot-assisted, 86 frame-based) undergoing surgery within 72 h of supratentorial basal ganglia hemorrhage was conducted. Propensity score matching balanced baseline characteristics between 40 patients per group. Results showed robot-assisted surgery achieved a significantly higher median hematoma evacuation rate (78.7% vs. 66.2%) and shorter median hospital stay (12 vs. 15 days) compared to frame-based surgery, with no significant differences in residual hematoma volume, surgical time, postoperative complications, or short-term functional outcomes. While robot-assisted techniques enhance evacuation efficiency and reduce hospitalization without increasing risks, their long-term neurological benefits require further investigation. These findings highlight the potential of robotic assistance as a safe and effective minimally invasive approach for ICH management.

## Introduction

Intracerebral hemorrhage (ICH) is a critical condition characterized by bleeding within the brain parenchyma, leading to high rates of mortality and morbidity^1^. Prompt and effective evacuation of the hematoma is essential to alleviate intracranial pressure and prevent secondary brain injury^2^. Compared to craniotomy, minimally invasive surgery (MIS) minimizes damage to healthy brain tissue^3,4^. Multiple studies have shown that MIS is associated with lower mortality rates, reduced risk of rebleeding and complications, and more favorable clinical outcomes^5^. As a result, there is a clear enthusiasm for the use of MIS techniques in the acute treatment of moderate to large intracerebral hemorrhages^6^. Frame-based stereotactic techniques, as traditional MIS, have been used to facilitate hematoma evacuation, offering precision in targeting deep-seated lesions. However, these methods can be cumbersome, time-consuming, and may pose challenges in terms of patient comfort and procedural flexibility.

In recent years, the advent of robot-assisted stereotactic systems has introduced new options for MIS in the treatment of ICH. It offers high localization accuracy and strong resistance to interference, making it a promising direction for the future development of minimally invasive strategies for ICH^7^. A meta-analysis indicated that robot-assisted MIS outperforms traditional surgery or conservative treatment in terms of rebleeding rates, neurological improvement, and the incidence of intracranial infections^8^. A study published in 2024 showed that compared to traditional manual puncture, stereotactic robot-guided puncture for hematoma drainage offers shorter operative time, less tissue damage, reduced inflammatory response, higher hematoma clearance efficiency, and improved neurological recovery^9^.

Despite these advancements, comprehensive comparative analyses between robot-assisted and traditional frame-based stereotactic approaches for ICH evacuation remain limited. This study aims to evaluate the efficacy and safety of robot-assisted stereotactic intracerebral hematoma evacuation in comparison to the conventional frame-based method.

## Methods

### Patient selection

A retrospective review was conducted on patients with ICH who underwent either frame-based or robot-assisted stereotactic surgery at the Neurosurgery Department of Ningbo Medical Center Lihuili Hospital between January 2022 and December 2024. Inclusion criteria were as follows: (1) age between 18 and 80 years; (2) surgical intervention performed within 72 h of intracerebral hemorrhage onset; (3) supratentorial basal ganglia hemorrhage; (4) hematoma volume of at least 20 mL within the brain parenchyma, excluding intraventricular hemorrhage from the volume calculation. Exclusion criteria were as follows: (1) incomplete patient history, laboratory results, or imaging data; (2) ICH secondary to cerebrovascular malformations or brain tumors; (3) involvement of thalamic, cerebellar, or brainstem hemorrhage; and (4) conversion to craniotomy due to excessive intraoperative bleeding during the surgery.

### Data collection

**Fig. 1:**
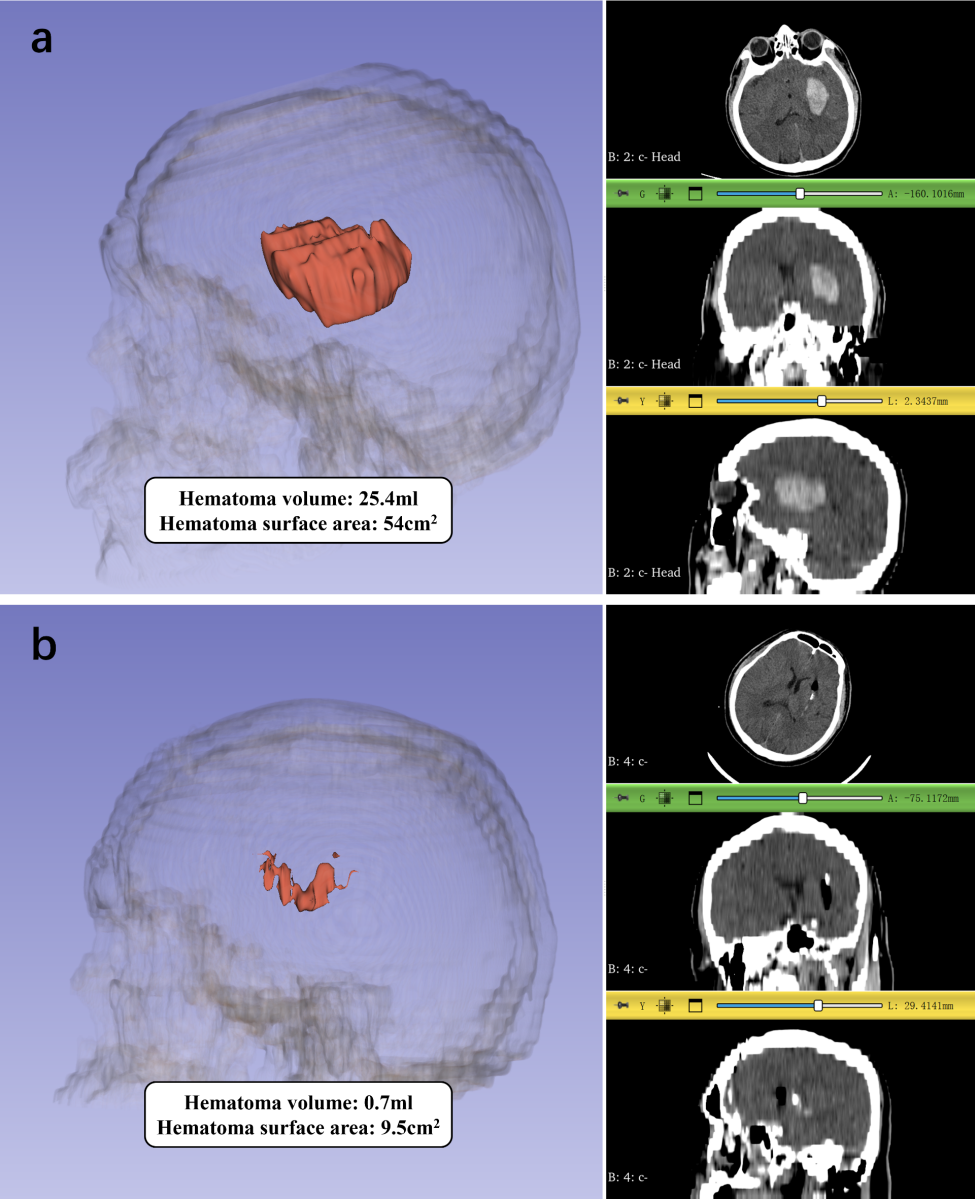
Hematoma volume and surface area assessment with 3D slicer (**a**: Pre-operation,** b**: Post-operation).

Demographic and clinical variables were collected, including sex, age, systolic and diastolic blood pressure upon emergency room admission, history of diabetes, and prior use of anticoagulant or antiplatelet medications. Additionally, preoperative Glasgow Coma Scale (GCS) scores and onset-to-surgery time were recorded. Laboratory parameters retrieved from the hospital database included blood type, hemoglobin levels, neutrophil and lymphocyte counts, platelet count, prothrombin time (PT), and activated partial thromboplastin time (APTT). Hematoma volume and surface area were quantified using 3D Slicer (version 5.6.2; Slicer Community, Brigham and Women’s Hospital, Boston, MA, USA) based on the final preoperative CT scan, with intraventricular hemorrhage excluded from the calculation. Hematomas were manually segmented on a slice-by-slice basis using a threshold range of 50–100 Hounsfield units, followed by the generation of a three-dimensional model, enabling precise measurement of hematoma volume and surface area. Similarly, residual hematoma volume was assessed from the immediate postoperative CT (Fig. 1). Hematoma evacuation rate is defined as (preoperative hemorrhage volume - residual hematoma volume)/preoperative hemorrhage volume*100%. Surgical time is defined as the duration from skin incision to wound closure. If additional procedures, such as external ventricular drainage or other surgeries, were performed intraoperatively, the time required for these additional procedures was not included in the surgical time calculation. Rebleeding was defined as a postoperative hematoma volume greater than the preoperative volume, a postoperative hematoma volume less than the preoperative volume but with a difference of less than 5 mL, or a gradual increase in postoperative hematoma volume by 10 mL. Additionally, we distinguished residual hematoma by reviewing the surgeons’ operative reports (regarding intraoperative hematoma evacuation) and assessing hematoma morphology on pre- and postoperative CT scans^10^. Intracranial infection was diagnosed based on the following criteria: (1) Clinical symptoms such as postoperative fever, headache, or neck stiffness. (2) Abnormal cerebrospinal fluid (CSF) findings: WBC > 10 × 10⁶/L, glucose < 2.25 mmol/L, chloride < 120 mmol/L, and protein > 0.45 g/L. (3) Positive CSF bacterial culture. Patients with criteria 3 could be diagnosed individually. Patients with negative CSF culture result but positive for the first 2 diagnostic criteria were also diagnosed as having intracranial infection^11^. Tracheostomy was performed in patients requiring prolonged mechanical ventilation (≥ 7 days), those with severe airway protection impairment due to decreased consciousness, or those with significant pulmonary complications^12^.

### Surgical procedure

All surgeries in this study were performed under general anesthesia by an associate chief neurosurgeon with 12 years of experience and two senior attending neurosurgeons with 10 years of experience each.

Robot group: The procedures were performed using the Remebot system (RM-50; Bohui Weikang Technology Co., Ltd., Beijing, China). The system achieves submillimeter accuracy (≤ 0.5 mm). Preoperative CT or MRI data are used to generate a 3D reconstruction, enabling precise trajectory planning. The patient was placed in a supine position, with the head was fixed by skull clamp. The entire cranial stabilization system was then connected to the telescopic support arm of the Remebot. Automated laser facial scanning was employed to register preoperative cranial CTA data. The target point was designated at the center of the hematoma, while the entry point was selected near the ipsilateral Kocher’s point or the nearest non-eloquent cortex to the hematoma. During the procedure, hematoma aspiration was performed using a 10 mL syringe along the planned trajectory. The aspiration process was conducted at multiple depths and angles, with adjustments to the needle’s depth or orientation made when resistance was encountered. The hematoma cavity was irrigated with saline until the effluent was clear. Subsequently, a catheter was introduced along the puncture path into the residual hematoma cavity.

Frame group: The procedures were performed using the Anke stereotactic frame system (Shenzhen Anke High-tech Co., Ltd., Shenzhen, China). Under local anesthesia, the Anke headframe was secured to the patient, followed by a thin-slice cranial CT scan to precisely localize the hematoma. The patient was then transferred to the operating room and placed under general anesthesia. The design of the puncture trajectory and the hematoma aspiration technique were similar to those used in the robot-assisted group.

### Statistical analysis

Statistical analyses were conducted using SPSS (version 27.0; IBM Corporation, Armonk, NY, USA), with a significance threshold set at *P* < 0.05. The Kolmogorov–Smirnov test was applied to assess the normality of continuous variables. Normally distributed continuous variables were expressed as mean ± standard deviation (SD) and compared using the independent t-test, while non-normally distributed data were presented as median [interquartile range (IQR)] and analyzed using the Mann-Whitney U test. Categorical variables were reported as numbers (percentages). The Mann-Whitney U test was employed for ordered categorical variables, whereas Pearson’s chi-square test, continuity correction chi-square test, or Fisher’s exact test was used for unordered categorical variables, as appropriate. To mitigate potential imbalances in baseline characteristics between the two groups, propensity score matching (PSM) was performed using R software (version 4.3.2; R Foundation for Statistical Computing, Vienna, Austria).

### Ethics approval and consent to participate

This study was performed in accordance with the Declaration of Helsinki of the World Medical Association, and all procedures were carried out in accordance with the approved guidelines and regulations. This study was approved by our institutional ethics committee (Ningbo Medical Center Lihuili Hospital, Approval Number: KY2024SL506). Participant data were retrospectively reviewed and deidentified. Because of anonymization, informed consent was waived by the ethics committee of Ningbo Medical Center Lihuili Hospital.

## Results

### Characteristics of the patients

This study included 131 patients, with a mean age of 53 years and a male predominance (102 patients, 77.9%). The median hematoma volume was 30.5 mL. Among them, 45 patients underwent robot-assisted stereotactic surgery, while 86 underwent frame-based stereotactic surgery. The two groups showed no significant differences in demographics, clinical history, or most laboratory parameters (Table 1). However, the robot-assisted group had a lower neutrophil-to-lymphocyte ratio (*P* = 0.047) and a shorter prothrombin time (*P* < 0.001). Besides, the hematoma volume appeared smaller in the robot-assisted group than in the frame-based group, although the difference did not reach statistical significance (*P* = 0.130).

**Table 1:** Characteristics and clinical data of the patients.

Variables	Robot group (*n* = 45)	Frame group (*n* = 86)	*P*-value
Demographics			
Male sex, n(%)^‡^	38(84.4)	64(74.4)	0.189
Age (y), mean ± SD ^#^	54 ± 13	53 ± 12	0.681
Clinical history			
SBP(mmHg), mean ± SD ^#^	169 ± 24	168 ± 26	0.799
DBP(mmHg), mean ± SD ^#^	98 ± 18	99 ± 20	0.727
Diabetes mellitus, n(%)^§^	3(6.7)	8(9.3)	0.853
Anticoagulant/antiplatelet, n(%)^§^	2(4.4)	8(9.3)	0.517
GCS, median[IQR] ^†^	9[7–14]	10[8–13]	0.876
Laboratory test			
Blood type, n(%)^§^			0.472
Type A	9(20.0)	24(27.9)	
Type B	14(31.1)	18(20.9)	
Type O	17(37.8)	37(43.0)	
Type AB	5(11.1)	7(8.1)	
Hemoglobin, mean ± SD ^#^	143 ± 17	142 ± 20	0.912
NLR, median[IQR] ^†^	5.9[3.0–11.0]	8.3[4.3–14.8]	0.047**
Platelets, median[IQR] ^†^	207[166–245]	204[162–246]	0.915
PT, mean ± SD ^#^	11.0 ± 0.8	11.7 ± 1.1	< 0.001**
APTT, mean ± SD ^#^	29.0 ± 3.1	29.1 ± 3.2	0.913
Hematoma characteristics			
Left hemisphere, n(%)^‡^	22(48.9)	44(51.2)	0.805
IVH, n(%)^‡^	18(40.0)	26(30.2)	0.261
Volume(ml), median[IQR] ^†^	29.0[23.0-35.2]	33.3[25.5–44.0]	0.130
Surface area(cm^2^), median[IQR] ^†^	68.0[56.5–86]	73.0[58.8–98.5]	0.274
Onset-to-surgery time, n(%)^†^			0.494
6–12 h	22(48.9)	33(38.4)	
12–18 h	9(20.0)	21(24.4)	
18–24 h	2(4.4)	13(15.1)	
1–2days	10(22.2)	12(21.8)	
2–3days	2(4.4)	4(4.7)	

SBP, Systolic Blood Pressure; DBP, Diastolic Blood Pressure; SD, Standard Deviation; IQR, interquartile range; GCS, glasgow coma scale; NLR, Neutrophil-to-Lymphocyte Ratio; PT, prothrombin time; APTT, activated partial thromboplastin time; IVH, Intraventricular hemorrhage;

# t-test; † Mann-Whitney U test; ‡ Pearsons chi-square test; § Continuous correction chi-square test.

** *P* < 0.05.

### Propensity score matching (PSM) analysis

**Fig. 2:**
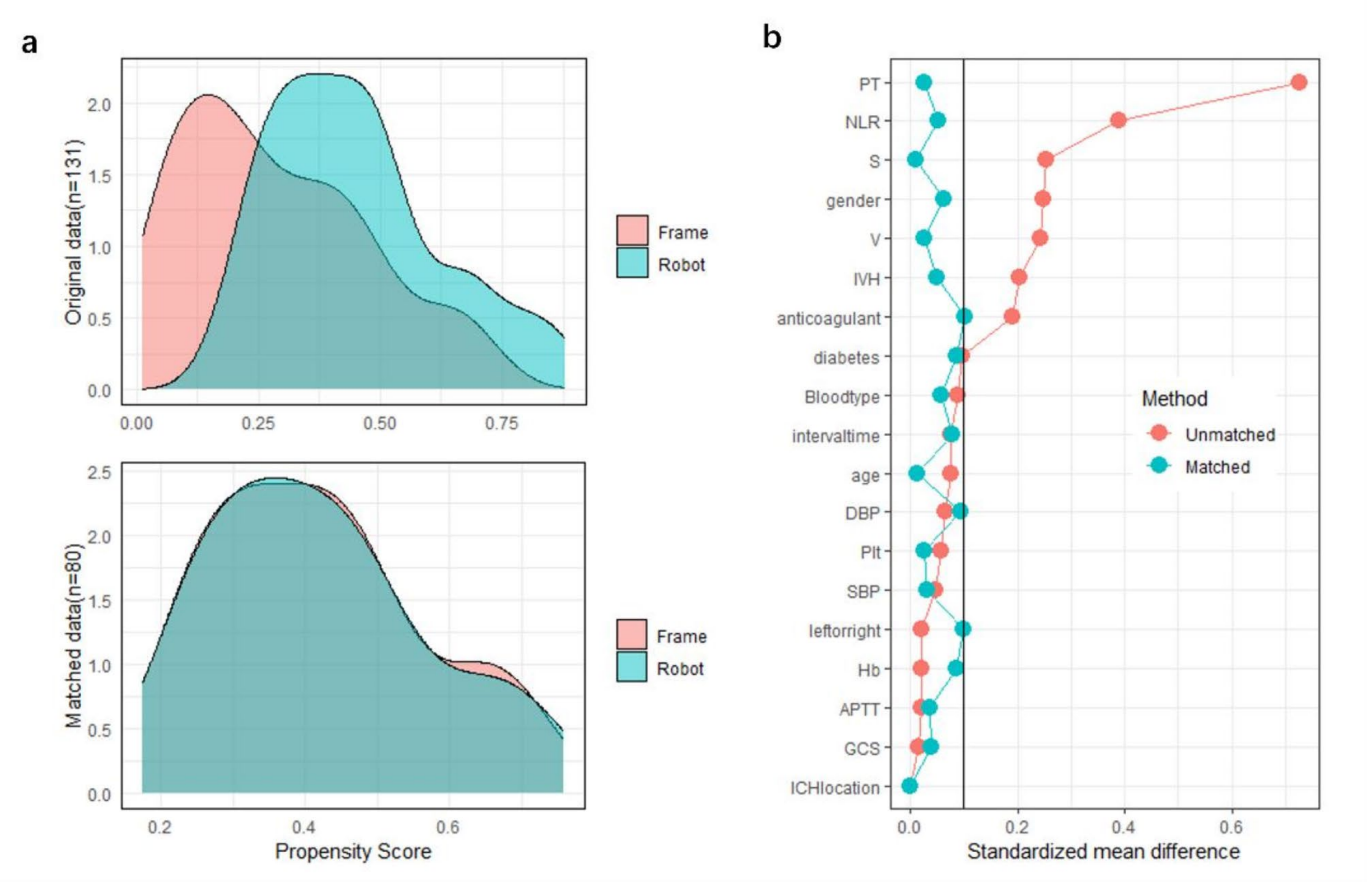
Propensity score distributions (**a**) and standardized mean differences (**b**) after PSM.

All variables in Table 1 were matched in a 1:1 ratio using a caliper width of 0.2. Propensity scores were estimated using logistic regression, and nearest neighbor matching was applied for case selection. After PSM, 40 patients were included in each group. The propensity score distributions were comparable between the two groups (Fig. 2a), and the standardized mean differences were significantly reduced compared to the pre-matching state (Fig. 2b). All variables, including hematoma volume (*P* = 0.874), were balanced between the two groups, with no significant differences in demographics, clinical history, laboratory parameters, or hematoma characteristics (Table 2).

**Table 2:** Characteristics and clinical data of the patients after propensity score matching.

Variables	Robot group (*n* = 40)	Frame group (*n* = 40)	*P*-value
Demographics			
Male sex, n(%)^‡^	33(82.5)	32(80.0)	0.775
Age (y), mean ± SD ^#^	53 ± 13	53 ± 12	0.956
Clinical history			
SBP(mmHg), mean ± SD ^#^	168 ± 24	167 ± 25	0.886
DBP(mmHg), mean ± SD ^#^	98 ± 18	96 ± 16	0.677
Diabetes mellitus, n(%)^§^	3(7.5)	4(10.0)	1.000
Anticoagulant/antiplatelet, n(%)^§^	2(5.0)	3(7.5)	1.000
GCS, median[IQR] ^†^	9[7–14]	11[7–13]	0.865
Laboratory test			
Blood type, n(%)^$^			0.208
Type A	8(20.0)	8(20.0)	
Type B	14(35.0)	9(22.5)	
Type O	14(35.0)	22(55.0)	
Type AB	4(10.0)	1(2.5)	
Hemoglobin, mean ± SD ^#^	144 ± 18	142 ± 16	0.700
NLR, median[IQR] ^†^	6.1[3.3–11.7]	7.4[3.9–10.0]	0.920
Platelets, median[IQR] ^†^	200[163–236]	197[161–234]	0.814
PT, mean ± SD ^#^	11.1 ± 0.7	11.1 ± 0.8	0.907
APTT, mean ± SD ^#^	29.1 ± 3.3	29.2 ± 3.2	0.871
Hematoma characteristics			
Left hemisphere, n(%)^‡^	21(52.5)	19(47.5)	0.655
IVH, n(%)^‡^	15(37.5)	16(40.0)	0.818
Volume(ml), median[IQR] ^†^	29.1[23.3–37.3]	29.3[23.4–40.5]	0.874
Surface area(cm^2^), median[IQR] ^†^	68.0[57.0–91.0]	67.0[57.3–90.3]	0.897
Onset-to-surgery time, n(%)^†^			0.524
6–12 h	20(50.0)	15(37.5)	
12–18 h	8(20.0)	10(25.0)	
18–24 h	1(3.5)	6(15.0)	
1–2days	9(22.5)	9(22.5)	
2–3days	2(5.0)	0(0.0)	

SBP, Systolic Blood Pressure; DBP, Diastolic Blood Pressure; SD, Standard Deviation; IQR, interquartile range; GCS, glasgow coma scale; NLR, Neutrophil-to-Lymphocyte Ratio; PT, prothrombin time; APTT, activated partial thromboplastin time; IVH, Intraventricular hemorrhage;

# t-test; † Mann-Whitney U test; ‡ Pearsons chi-square test; § Continuous correction chi-square test; $ Fisher’s Exact Test.

### Comparison of clinical outcomes after PSM

After PSM, the robot-assisted group showed a significantly higher median hematoma evacuation rate (78.7% vs. 66.2%, *P* = 0.032) and shorter median hospital stay (12 vs. 15 days, *P* = 0.001) compared to the frame-based group. Although residual hematoma volume (5.8 vs. 8.8 mL, *P* = 0.077) favored the robot-assisted group, this difference was not statistically significant. Surgical time and postoperative complications, including rebleeding, infection, and tracheostomy, as well as modified Rankin Scale (mRS) scores at discharge, were comparable between the two groups. (Table 3)

**Table 3:** Comparison of efficacy indicator and complications after propensity score matching.

Variables	Robot group (*n* = 40)	Frame group (*n* = 40)	*P*-value
Efficacy indicators			
Residual hematoma(ml), median[IQR] ^†^	5.8[2.3–10.9]	8.8[5.0-18.2]	0.077
Hematoma evacuation rate(%), median[IQR] ^†^	78.7[69.7–91.8]	66.2[48.1–88.2]	0.032**
Surgical time(min), mean ± SD ^#^	84 ± 13	80 ± 16	0.151
Postoperative complications, n(%)^‡^	10(25.0)	16(40.0)	0.152
Rebleeding, n(%)^§^	3(7.5)	6(15.0)	0.479
Intracranial infection, n(%)^$^	0(0.0)	1(2.5)	1.000
Tracheostomy, n(%)^‡^	8(20.0)	10(25.0)	0.592
Hospitalization and outcome			
Hospital stay(days), median[IQR] ^†^	12[8–15]	15[13–20]	0.001**
mRS Score at discharge, n(%)^†^			0.619
6	0(0.0)	0(0.0)	
5	11(27.5)	10(25.0)	
4	24(60.0)	23(57.5)	
3	4(10.0)	6(15.0)	
2	1(2.5)	1(2.5)	
1	0(0.0)	0(0.0)	

SD, Standard Deviation; IQR, interquartile range. # t-test; † Mann-Whitney U test; ‡ Pearsons chi-square test; § Continuous correction chi-square test; $ Fisher’s Exact Test. ** *P* < 0.05.

## Discussion

ICH remains a major cause of morbidity and mortality, necessitating prompt hematoma evacuation while minimizing edema and surgical trauma. MIS offers a promising approach to achieving these goals^13^. However, the MISTIE III trial failed to demonstrate a significant functional outcome benefit^14^. Several factors, including mechanical trauma to adjacent brain tissue during surgical manipulation, limited intraoperative visualization, catheter deviation, and a larger residual hematoma, contribute to suboptimal postoperative neurological recovery^15,16^.

Some studies suggest that robot-assisted stereotactic surgery, as a form of MIS, may have the potential to enhance treatment in ICH management^17^. A study published in 2024 found that, compared to traditional craniotomy, robot-assisted stereotactic surgery can shorten surgery and hospital stay times, reduce complication rates, and improve patient outcomes^18^. Another study showed that robotic-assisted stereotactic surgery for intracerebral hemorrhage offers health economic benefits, with reduced costs and slightly better outcomes compared to neuro-endoscopic surgery^19^. However, there are limited studies comparing robot-assisted stereotactic surgery with traditional frame-based techniques for ICH evacuation^20,21^. A recent meta-analysis of 17 studies identified only one study that directly compared these two approaches^22^. In this study, we employed PSM to rigorously assess their efficacy and safety.

This study utilized the Remebot system, the first neurosurgical robot approved by China’s National Medical Products Administration. The system features a six-degree-of-freedom robotic arm, an optical tracker for registration, and specialized planning and navigation software, achieving submillimeter accuracy (≤ 0.5 mm). Preoperative CT or MRI data are used to generate a 3D reconstruction, enabling precise trajectory planning, while the robotic arm automatically aligns with the predefined trajectory to ensure accuracy^23^. Unlike the ROSA system (Zimmer Biomet, USA), which is widely used globally for various neurosurgical procedures such as epilepsy surgery and electrode implantation, the Remebot system is primarily utilized in China and is more specialized in hematoma drainage and neurosurgical puncture-related applications. A 2019 study reported 17 cases of ICH treated with the Remebot system. The mean targeting error was 1.28 ± 0.49 mm, with hematoma reduction rates ranging from 70 to 90%. At the three-month follow-up, 58.8% of patients had resumed daily activities, and postoperative complications were minimal^24^.

### Efficacy indicators

Given the substantial estimation error inherent in the ABC/2 formula for calculating hematoma volume, particularly in cases involving multilobular hematomas where the error can be as high as 39%^25^, this study utilized 3D Slicer to obtain more precise measurements of hematoma volume, especially for irregularly shaped residual hematomas post-surgery. Besides, previous studies have suggested that the irregularity of hematoma shape can affect the efficacy of MIS and the prognosis of patients with intracerebral hemorrhage^15,26^. However, the assessment of hematoma irregularity remains subjective. To improve the objectivity of evaluating hematoma morphology, we used hematoma surface area as a quantitative measure. Additionally, we incorporated hematoma surface area as a matching factor in the PSM process to minimize potential biases.

In this study, the robot-assisted group achieved a median evacuation rate of 78.7%, significantly higher than the 66.2% observed in the frame-based group. Although no studies have directly compared these two approaches, prior literature reports a 77% evacuation rate for robot-assisted surgery^27^, consistent with our finding, while frame-based surgery has been reported with rates ranging from 41–75%^28–32^. Although the residual hematoma volume was smaller in the robot-assisted group (5.8 mL vs. 8.8 mL), the difference did not reach statistical significance (*p* = 0.077). This trend suggests that robotic assistance may facilitate more complete hematoma removal, potentially reducing secondary injury. Given that both groups employed similar aspiration techniques, the superior clearance rate in the robot-assisted group likely reflects the system’s improved trajectory planning and intraoperative accuracy. A study suggested that robotic systems, free from frame constraints, provided a larger surgical workspace and utilized 3D reconstruction to enhance hematoma visualization, enabling more precise trajectory planning and improved evacuation^33^.

Surgical time did not differ significantly between the two groups, with the robot-assisted group averaging 84 ± 13 min and the frame-based group 80 ± 16 min. However, a study published in 2022 reported significantly shorter surgical times of 29 ± 8 min and 41 ± 9 min, respectively^33^. The prolonged surgical time in our study may be attributed to the additional time spent irrigating the hematoma cavity with saline until the effluent became clear, a practice we believe may help reduce the risk of rebleeding. Similarly, a 2024 study on ROSA for intracerebral hematoma aspiration reported a time of 78 ± 24 min, close to our results^34^. In this study, surgical time was measured from skin incision and did not include preoperative preparation time, which may explain the lack of significant difference between the two groups. Notably, frame-based procedures require additional preoperative steps, including frame placement and localization CT, resulting in a much longer preparation time compared to robot-assisted surgery. However, a limitation of our study is that we did not precisely record the preoperative preparation time, preventing a direct comparison. Additionally, the need for localization CT in frame-based surgery increases both medical costs and radiation exposure for patients.

### Postoperative complications

In terms of postoperative complications, including rebleeding, infection, and tracheostomy, both groups had similar outcomes, suggesting that the robot-assisted technique does not introduce additional risks compared to the traditional method. However, a meta-analysis showed that robot-assisted surgery significantly reduced the risk of rebleeding compared to the frame-based surgery^21^. Further research is needed to validate this finding; however, our results indicate that robot-assisted surgery is a safe approach for hematoma evacuation.

### Hospitalization and outcome

Patients in the robot-assisted group had a shorter median hospital stay (12 days vs. 15 days in the frame-based group). This reduction in hospital stay may be attributed to the more efficient evacuation of the hematoma, leading to quicker recovery and less postoperative management. To date, no other studies have directly compared hospital stay durations between these two groups. One meta-analysis evaluating various surgical modalities reported that robot-assisted procedures significantly reduced hospital stay compared with craniotomy, whereas no significant differences were observed when compared with conservative treatment or endoscopic approaches^22^.

The mRS scores at discharge were comparable between the robot-assisted and frame-based groups (*p* = 0.619), with most patients exhibiting moderate to severe disability (mRS 4–5). No significant differences were observed, suggesting that while robot-assisted surgery may shorten hospital stay, its impact on short-term functional outcomes remains unclear. Since long-term follow-up was not conducted in this study, the potential effects of robot-assisted surgery on long-term neurological recovery remain uncertain.

### Limitations

This study has several limitations. First, its retrospective design introduces potential selection bias, despite the application of propensity score matching to minimize confounding variables. Second, the relatively small sample size may limit the statistical power to detect differences in certain outcomes, such as residual hematoma volume and mRS scores at discharge. Larger multicenter studies are needed to validate these findings. Third, robot-assisted surgery was predominantly applied in the later cases of our cohort, during which improvements in the surgeons’ hematoma aspiration techniques may have contributed to the observed outcomes. Fourth, due to the limited sample size, surgeon experience was not included in the propensity score matching analysis, and differences in postoperative management were not systematically accounted for. These factors may introduce additional confounders, and future studies should consider adjusting for intraoperative and postoperative management differences. Fifth, this study did not include long-term follow-up data, preventing an evaluation of the potential impact of robot-assisted surgery on long-term functional recovery. Future prospective studies with extended follow-up are necessary to determine whether the observed advantages in hematoma evacuation and hospital stay translate into improved long-term neurological outcomes. Finally, the lack of cost-related data is another limitation, and future studies should explore the economic impact of robotic-assisted hematoma evacuation.

## Conclusions

This study demonstrates that robot-assisted stereotactic surgery achieves a significantly higher hematoma evacuation rate and shorter hospital stay compared to the conventional frame-based approach, without increasing the risk of postoperative complications. While its impact on long-term outcomes remains unclear, further prospective studies are needed to confirm its clinical benefits.

## Data Availability

All data are available within the text of the article. Further anonymized data could be made available to qualified investigators upon reasonable request by contacting the corresponding author.

## Abbreviations

ICH: Intracerebral hemorrhage
MIS: Minimally invasive surgery
GCS: Glasgow Coma Scale
PSM: Propensity score matching
PT: Prothrombin time
APTT: Activated partial thromboplastin time
SD: Standard deviation
IQR: Interquartile range
